# Reflection on “Site-specific PEGylation of proteins by a Staudinger-phosphite reaction”: from protein modification to ADCs in the clinic

**DOI:** 10.1039/d6sc90102f

**Published:** 2026-05-19

**Authors:** Karl T. Schuppe, Christian P. R. Hackenberger

**Affiliations:** a Leibniz-Forschungsinstitut für Molekulare Pharmakologie (FMP), Department Chemical Biology Robert-Rössle-Straße 10 13125 Berlin Germany hackenbe@fmp-berlin.de; b Humboldt-Universität zu Berlin, Department of Chemistry Brook-Taylor-Straße 2 12489 Berlin Germany

## Abstract

Chemoselective or bioorthogonal modification reactions resulted in several breakthrough studies that enabled the incorporation of functional modules into proteins and antibodies for basic and translational research. In 2010, we published a paper in *Chemical Science*, which described a chemoselective method for synthesizing branched PEGylated peptides and proteins using the Staudinger-phosphite reaction (R. Serwa, P. Majkut, B. Horstmann, J. M. Swiecicki, M. Gerrits, E. Krause and C. P. R. Hackenberger, *Chem. Sci.*, 2010, **1**, 596–602, https://doi.org/10.1039/C0SC00324G). We discuss subsequent studies in using the protocol for the intracellular stabilization of peptides and the development of the P5-labeling platform, which we currently use in the generation of antibody–drug-conjugates (ADCs) as next-generation biopharmaceuticals in clinical studies, for which a first proof-of-concept study was also published in *Chemical Science* (P. Ochtrop, J. Jahzerah, P. Machui, I. Mai, D. Schumacher, J. Helma, M. A. Kasper and C. P. R. Hackenberger, *Chem. Sci.*, 2023, **14**, 2259–2266, https://doi.org/10.1039/D2SC05678J).

## Abstract 2

In celebration of our 15th anniversary and some of our most popular articles, Christian Hackenberger reflects on the area of site-specific protein bioconjugation, covering developments since a paper in Chemical Science on this topic: https://doi.org/10.1039/C0SC00324G.

## Graphical abstract

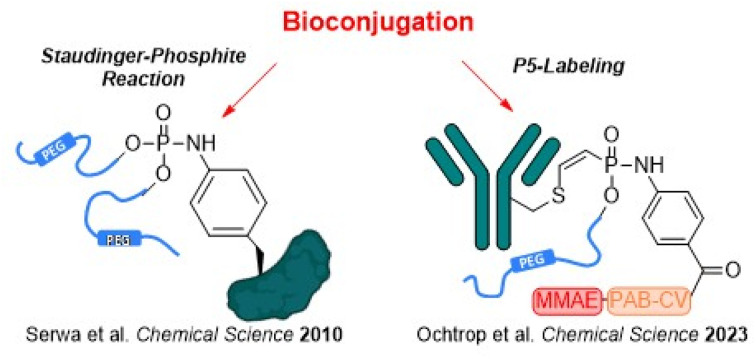

Polyethylene glycol (PEG) has long been valued in chemical biology and pharmaceutical chemistry for its capacity to enhance the solubility, stability, and pharmacokinetic properties of biomolecules.^1^ PEGylation has therefore become a widely used strategy in the development of therapeutic agents,^2^ especially in the context of protein bioconjugation.^3^

Shortly after our first report of using the Staudinger-phosphite reaction^4^ for the chemoselective modification of proteins,^5,6^ our group used this strategy for PEGylating azide-containing peptides and proteins. In this transformation, published in *Chemical Science* in 2010, a phosphite carrying three PEG-moieties reacts with an azide-bearing peptide to form a phosphoramidate linkage (Fig. 1).^7^ The resulting phosphorus center bears two linear PEG chains and thus acts as a branching point, enabling branched PEG architectures that can provide greater stabilization than linear polymer chains through the so-called “umbrella effect”.^3,7^ Importantly, the transformation proceeds efficiently under biologically relevant conditions, including physiological pH and even in crowded environments such as crude cell lysates. To further expand the method, we also introduced a photolabile nitrobenzyl-derivative between the phosphorus center and the PEG chain, enabling light-triggered de-PEGylation.^7^

**Fig. 1:**
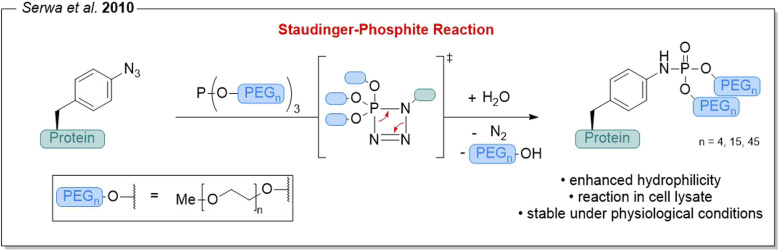
PEGylation of azide-containing proteins *via* the Staudinger-phosphite reaction, forming a phosphoramidate linkage using a PEG-substituted phosphite.^7^

After establishing the reliability and chemoselectivity of this PEGylation strategy, we examined its biological impact. Apoptosis-inducing BH3 peptides modified with solubilizing PEG chains using this approach showed enhanced hydrophilicity, increased half-life, and improved apoptotic activity in Jurkat cells, along with a homogeneous cytoplasmic distribution. These findings highlighted the advantages of employing phosphoramidate-PEG-linkages in the generation of bioactive peptides with pharmacological potential.^8^

In the years that followed, we broadened the scope of phosphorus-based conjugation reactions,^9^ in particular the Staudinger-phosphite reaction using phosphonites instead of phosphites in the reaction with azides.^10^ Furthermore, we developed reagents by integrating unsaturated alkynes and alkenes into their design.^11,12^ This strategic expansion enabled us to use the Staudinger-phosphonite reaction to generate a new class of conjugation handles with high selectivity for native cysteine residues.^13^ These advances ultimately laid the groundwork for what became the P5-labeling platform, a powerful tool for the conjugation and modification of biomolecules and a more stable alternative to the widely used maleimide strategy (Fig. 2).^14–17^ This is particularly relevant in the field of antibody–drug conjugates (ADCs), where maleimide-based conjugation is commonly employed despite several known limitations such as thiol exchange, retro-Michael instability, payload loss in circulation, and associated off-target toxicity.^13^ The strategy to introduce PEG into biomolecules therefore evolved alongside our advances in phosphorus chemistry. By using P5 conjugation handles, PEG could be incorporated as an alkoxy residue, allowing its addition simultaneously with the functional conjugation of a biomolecule.^14^

**Fig. 2:**
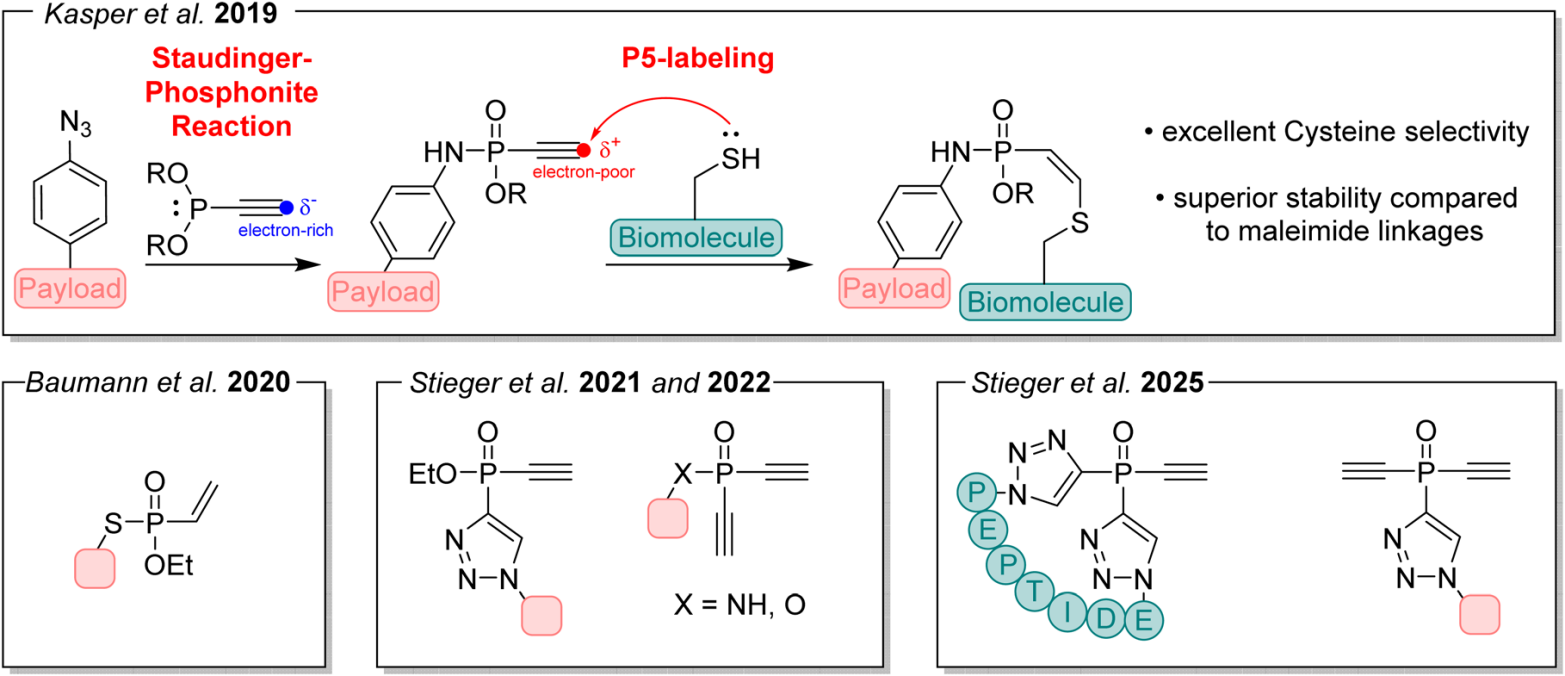
Synthesis of a P5-electrophile (ethynyl-phosphonamidate) followed by cysteine-selective conjugation. Various P5 reagents developed subsequently are shown underneath.^8,14–17^

Antibody–drug conjugates (ADCs), inspired by Paul Ehrlich's vision of “magic bullets” for chemotherapy, represent a rapidly growing class of targeted cancer therapies.^18,19^ As the name suggests, ADCs consist of a potent cytotoxic payload that is bound *via* a linker to a monoclonal antibody that specifically targets antigens on the surface of cancer cells. These biopharmaceuticals aim to combine the advantages of both components; the precise specificity of these protein molecules with the cytotoxic potency of chemotherapy. The field has become increasingly dynamic, with 15 ADCs now approved by the FDA, including three blockbuster drugs: Kadcyla (Roche), Adcetris (Seagen), and Enhertu (Daiichi Sankyo).^20^ Today, ADCs are no longer merely a conceptual postulate but have evolved into precision-guided chemotherapeutics that combine extreme potency with molecular accuracy. They offer an expanded therapeutic window, the potential to overcome resistance mechanisms, and a modular platform that can be rationally tailored to different tumor types.^21^

The combination of a water-soluble antibody with hydrophobic cytotoxic small molecules is an inherent problem that leads to aggregation, precipitation, off-target effects, and accelerated clearance from systemic circulation. To counterbalance the hydrophobicity of the payload, PEG chains can be built into the construct to improve its physicochemical properties. This also opens the possibility to attach more payload molecules to the antibody, increasing the conjugate's potency.^19^ However, when PEG chains are used as a linear spacer between the antibody and payload, they can increase unwanted solvent exposure, promoting nonspecific hydrophobic interactions.^22^

In our 2023 publication, also published in *Chemical Science*, we directly incorporated a PEG sidechain into our P5-labeling reagent, combining our previous PEGylation strategy with the efficiency and stability of the P5-labeling platform for cysteine-selective bioconjugation of antibodies (Fig. 3).^23^ We used this approach to conjugate brentuximab with MMAE *via* a valine–citrulline *p*-aminobenzylcarbamate (VC-PAB) linker using an ethynyl phosphonamidate handle containing a PEG sidechain. This allowed us to increase the drug-antibody ratio (DAR) to up to eight, without affecting water solubility. These DAR 8-ADCs exhibited an excellent pharmacokinetic profile and stability *in vivo* and also showed higher potency, resulting from the increased loading of payloads, compared to the maleimide-based, unPEGylated ADC Adcetris.^23^

**Fig. 3:**
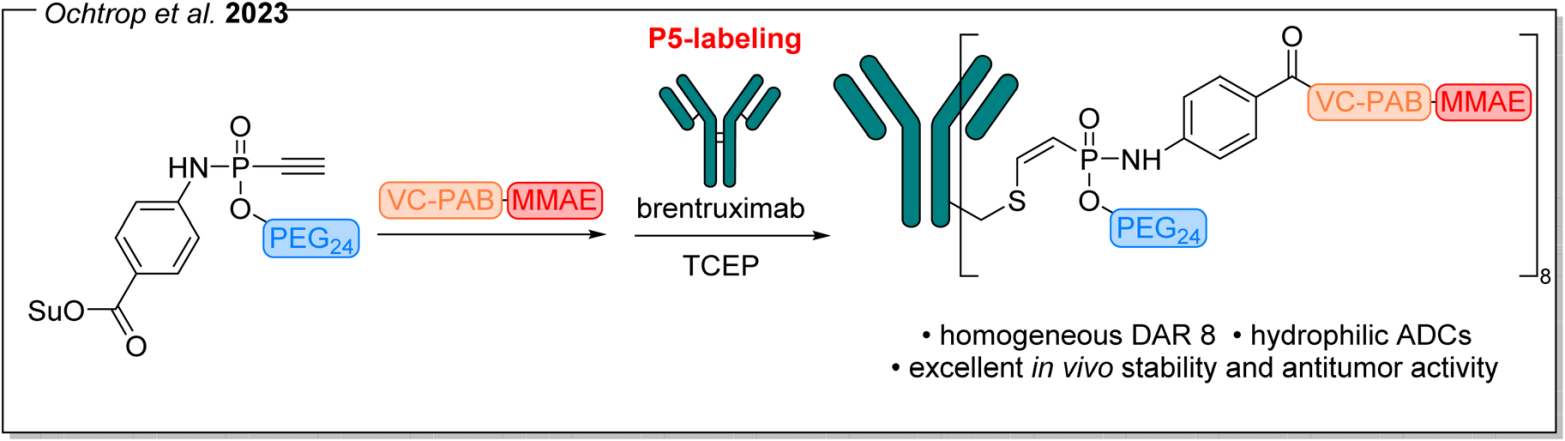
P5-labeling of brentuximab with MMAE using PEGylated ethynyl-phosphonamidates, enabling higher DARs and an improved hydrophilicity and *in vivo* efficacy compared to Adcetris®, an FDA-approved therapeutic ADC^23^ (Su = succinimide).

To translate these academic findings into therapeutics for oncology, the concept was further developed by the FMP and LMU spin-off Tubulis®, founded in 2019.^24^ Tubulis® developed the clinical ADC candidates TUB-030 and TUB-040, both employing the same P5-based conjugation handle that had previously been demonstrated to be efficient in our laboratory (Fig. 4). TUB-040 is being developed for the treatment of platinum-resistant ovarian cancer (PROC), while TUB-030 targets 5T4, a cancer-associated antigen expressed in a wide range of solid tumors. Both candidates are currently in Phase I/IIa clinical studies, and the first clinical interim data were presented in 2025 at the ESMO Congress.^24,25^

**Fig. 4:**
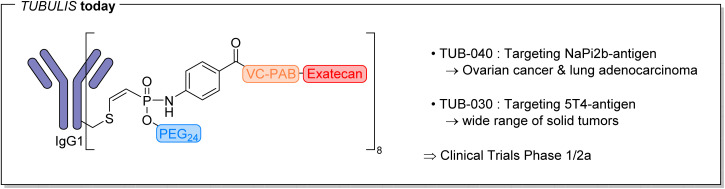
P5-based ADCs TUB-030 and TUB-040 in early clinical development for solid tumors and platinum-resistant ovarian cancer.^25^

The P5 story is far from complete, and ongoing research continues to yield new insights. The potential of this chemistry is far from exhausted. Our recent publications show that the P5 platform is versatile, modular and adaptive and can be used in the design of covalent inhibitors or cystein-directed protein targeting^26^ as well as the development of probes to target protein tyrosine phosphatases.^27^ To date, our efforts originated from transforming a classical organic reaction, namely the Staudinger reaction, into a chemoselective protein modification strategy and ultimately into a successful conjugation platform to obtain ADCs for cancer therapy, thereby illustrating a hallmark of chemical biology: developing new chemical tools to probe biological systems and advance therapeutic avenues for our society.

## Author contributions

K. T. S. and C. P. R. H. drafted and revised the manuscript jointly.

## Conflicts of interest

C. P. R. H. is a co-founder, shareholder and advisor of Tubulis GmbH and holds patents on P5-labeling.

## Data Availability

There is no additional data associated with this article.
